# A New Human SCARB2 Knock-In Mouse Model for Studying Coxsackievirus A16 and Its Neurotoxicity

**DOI:** 10.3390/v17030423

**Published:** 2025-03-14

**Authors:** Haiting Wu, Ziou Wang, Yiwei Zhang, Lingfeng Hu, Jinling Yang, Caixing Zhang, Mumeng Lou, Na Pi, Qiyan Wang, Shengtao Fan, Zhangqiong Huang

**Affiliations:** Institute of Medical Biology, Chinese Academy of Medical Sciences & Peking Union Medical College, No. 935, Jiaoling Road, Kunming 650118, China; wuht@student.pumc.edu.cn (H.W.); wangziou1226@163.com (Z.W.); zyw@student.pumc.edu.cn (Y.Z.); 13187715267@163.com (L.H.); yangjl0117@imbcams.com.cn (J.Y.); 2675740248@imbcams.com.cn (C.Z.); 459866462@163.com (M.L.); 2345655391@163.com (N.P.); qywang127@163.com (Q.W.)

**Keywords:** hand, foot and mouth disease, CVA16, hSCARB2 knock-in mice, CRISPR/Cas9, neuropathology

## Abstract

Hand, Foot, and Mouth Disease (HFMD) is a viral illness caused by enterovirus infections. While the introduction of the enterovirus 71 (EV71) vaccine has significantly reduced the number of EV71-related cases, the continued spread of Coxsackievirus A16 (CVA16) remains a major public health threat. Previous studies have shown that human SCARB2 (hSCARB2) knock-in (KI) mice, generated using embryonic stem cell (ESC) technology, are susceptible to CVA16. However, these models have failed to reproduce the clinical pathology and neurotoxicity after CVA16 infection. Therefore, there is an urgent need for a more reliable and effective animal model to study CVA16. In this study, we successfully created a hSCARB2 KI mouse model targeting the ROSA26 locus using CRISPR/Cas9 gene editing technology. The application of CRISPR/Cas9 enabled stable and widespread expression of hSCARB2 in the model. After infection, the KI mice exhibited a clinical pathology that closely mimics human infection, with prominent limb weakness and paralysis. The virus was detectable in multiple major organs of the mice, with peak viral load observed on day 7 post-infection, gradually clearing thereafter. Further analysis revealed widespread neuronal necrosis and infiltration of inflammatory cells in the brain and spinal cord of the KI mice. Additionally, significant activation of astrocytes (GFAP-positive) and microglia (IBA1-positive) was observed in the brain, suggesting that CVA16 infection may induce limb paralysis by attacking neuronal cells. Overall, this model effectively replicates the neuropathological changes induced by CVA16 infection and provides a potential experimental platform for studying CVA16-associated pathogenesis and neurotoxicity.

## 1. Introduction

Hand, foot, and mouth disease (HFMD) is a highly contagious viral illness that primarily affects children under the age of five [1]. The clinical manifestations of HFMD include fever and vesicular lesions on the hands, feet, and oral mucosa, with similar lesions occasionally observed on the buttocks in some cases [2]. Although HFMD is generally a self-limiting disease, a small proportion of patients may develop severe complications, such as meningitis, encephalitis, and acute flaccid paralysis; some severe complications can lead to death within 24–48 h [3,4]. Thus, HFMD poses a significant public health threat, with outbreaks predominantly occurring in the Asia-Pacific region [2,5,6]. The primary causative agents of HFMD belong to the Picornaviridae family, which includes enteroviruses and coxsackieviruses. Among these, coxsackievirus A16 (CVA16) and enterovirus 71 (EV71) are the major pathogens responsible for HFMD outbreaks [7]. However, specific antiviral drugs and effective treatments for HFMD remain under development [8]. Therefore, vaccination with safe and effective vaccines is one of the most efficient strategies for controlling HFMD spread.

In 2016, the introduction of the EV71 vaccine significantly reduced the number of HFMD cases caused by EV71 infection [5,9]. However, this vaccine does not provide cross-protection against other heterologous enteroviruses [10]. Since 2017, the majority of global HFMD outbreaks have been attributed to CVA16. Although it is clinically challenging to differentiate HFMD symptoms caused by different serotypes, studies have shown that children infected with CVA16 or coxsackievirus A10 (CVA10) exhibit a greater incidence of oral vesicles than those infected with EV71 [11]. Additionally, CVA16 infection can lead to severe complications, such as fatal myocarditis, pneumonia, and encephalitis, resulting in limited clinical treatment options. Central nervous system (CNS) diseases caused by CVA16 are often associated with high mortality rates [12].

In response to this growing public health challenge, it is imperative to establish effective animal models to facilitate the development of broader-spectrum and more efficient vaccines [13]. While nonhuman primate models can partially replicate the neurological complications observed in human clinical cases, their use is significantly constrained by ethical and economic limitations [14]. Existing mouse models for CVA16 infection rely primarily on the use of mouse-adapted viral strains, immunodeficient mice, or immunocompetent neonatal mice [15,16,17]. Unfortunately, the mutational characteristics of mouse-adapted viral strains, the influence of artificial viral inoculation routes, and interventions for immune deficiencies may fail to accurately reflect the natural pathogenesis of human disease. Moreover, owing to the small size of neonatal mice, experimental procedures pose significant technical challenges and these models cannot fully replicate the complete course and pathological effects of CVA16 infection [18,19]. Therefore, there is an urgent need to develop new experimental animal models to overcome the limitations of existing models and advance related research.

CVA16 infection results in host restriction, with humans being the only known natural host [20]. Studies have identified human scavenger receptor class B member 2 (hSCARB2) as a candidate cellular receptor for both CVA16 and EV71 [21,22], playing a critical role in the early stages of viral infection. SCARB2, also known as lysosomal integral membrane protein 2, is localized primarily to lysosomes and functions as a lysosomal targeting receptor for β-glucocerebrosidase [23,24]. Previously, researchers have used embryonic stem cell (ESC) technology to generate hSCARB2 knock-in (KI) mice, which are susceptible to CVA16. Viral replication was detectable in these mice and they exhibited some degree of weight loss. However, the model failed to replicate the neurological, clinical, and pathological symptoms of human HFMD [25,26]. Additionally, the process of generating KI mice via ESC technology is complex, costly, and associated with a high risk of off-target effects, which limits its practical application [27]. Therefore, we used CRISPR/Cas9 gene-editing technology in this study to construct a new hSCARB2 gene locus-specific protein knock-in chimeric mouse model to provide a new tool for studying the pathogenesis of CVA16, offering a more suitable and effective animal model for subsequently developing vaccines and therapeutic drugs.

## 2. Materials and Methods

### 2.1. Ethics Statement

The genomic editing and pathogen-related experiments were reviewed and approved by the Institutional Ethics Review Committee of the Institute of Medical Biology and all procedures were conducted in strict accordance with the Institute’s animal research guidelines. All tissue samples used in this study were obtained in an ethically sound manner and the study protocol was approved by the relevant ethics committee of the Institute of Medical Biology. The ethical approval number for the experimental animals was DWSP20240344.

### 2.2. Cell and Virus

Vero cells were purchased from ZQXZBIO (Shanghai, China) and maintained in Dulbecco’s Modified Eagle Medium (DMEM) supplemented with 10% FBS at 37 °C in a 5% CO_2_ humidified atmosphere. The CVA16 virus KM/M08 strain (GenBank: MN046208) was acquired from the Institute of Medical Biology.

### 2.3. Virus Enrichment and Titration

When Vero cells reached 80–90% confluence, they were inoculated with the CVA16 virus at a multiplicity of infection (MOI) of 0.1. The cells were incubated at 37 °C for 2 h to allow viral adsorption, after which 15 mL of virus maintenance medium (2% FCS) was added. The cultures were maintained in an incubator and the cytopathic effects (CPEs) were monitored at 8 h intervals. The virus was harvested when 80–90% of the cells in the culture flask presented a CPE. The supernatant was collected by centrifugation at 5000 rpm for 10 min to remove cellular debris. The clarified virus suspension was aliquoted into 1.5 mL EP tubes, with 500 µL per tube, and stored at −80 °C for subsequent experiments.

The confluent monolayer of Vero cells from a T75 flask was harvested and transferred to a 15 mL centrifuge tube. The cells were resuspended in DMEM supplemented with 4% FBS to prepare a single-cell suspension, and the concentration was adjusted to 1 × 10^5^ cells/mL using a cell counter. A 96-well plate was seeded with 100 μL of the cell suspension per well to allow cell attachment. The virus stock was then subjected to 10-fold serial dilutions in DMEM, ranging from 10^−1^ to 10^−10^, and 100 μL of each dilution was added to eight replicate wells per dilution. Control groups were included, with 100 μL of undiluted virus stock in the positive control wells and 100 μL of DMEM in the negative control wells. The plate was incubated at 37 °C with 5% CO_2_, and cytopathic effects (CPE) were observed every 8 h for 5–7 days until clear CPE development was noted for TCID_50_ calculation.

### 2.4. Generation of ROSA26-Targeted hSCARB2 KI F0 Mice via Cytoplasmic Microinjection

The C57BL/6J and ICR mice used in this study were purchased from the Laboratory Animal Experiment Department of the Institute of Medical Biology, Chinese Academy of Medical Sciences [Animal Production License No. SCXK (JING)2016-0006]. The animals were housed in a barrier environment under controlled conditions with a constant temperature of 20 ± 2 °C and a relative humidity of 50–60% [Animal Facility License No. SYXK (Dian) K2021–0001]. The mice had ad libitum access to food and water and were maintained on a 12 h light/dark cycle.

Single-guide RNAs (sgRNAs) were designed by Beijing Vitalstar Biotechnology Co., Ltd. (Beijing, China). The specific allele site was selected as the target sequence in intron 1 of the mouse ROSA26 locus. The target sequence was a sgRNA (CCGCGCGCCCCTGCGCAACG). The sgRNAs and Cas9 mRNA were produced via in vitro transcription (IVT). The plasmid pCAG-GFP-N1 was utilized as the backbone for constructing the targeting donor DNA. Initially, the left homologous arm (LHA) of the ROSA26 locus was ligated into pCAG-GFP-N1, successfully generating pCAG-GFP-N1-ROSA26 LHA. High-fidelity PCR was subsequently performed to amplify the ROSA26 right homologous arm (RHA), the hSCARB2 fragment, and the WPRE-polyA sequence, which were purified for downstream applications. The plasmid pCAG-GFP-N1-ROSA26 LHA was then digested with HindIII and the larger fragment was recovered to serve as the cloning vector. Finally, the three amplified fragments were integrated into the vector via the Gibson assembly method, successfully constructing the pmROSA26-CAG-hSCARB2 targeting vector.

Four-week-old female C57BL/6J mice were administered intraperitoneal injections (i.p.) of pregnant mare serum gonadotropin (PMSG, 10 U/mouse), followed by human chorionic gonadotropin (hCG, 10 U/mouse) 48 h later. Immediately after the hCG injection, the females were mated with 12-week-old male C57BL/6J mice. Fertilized embryos were collected the following morning. For fertilized eggs microinjection, a mixture containing Cas9 mRNA (10 ng/μL), sgRNA (5 ng/μL), and the hSCARB2-targeting construct (10 ng/μL) was injected into the fertilized eggs The surviving fertilized eggs were cultured at 37 °C with 5% CO_2_ for 2 h and subsequently selected at the two-cell stage for transfer into the oviducts of pseudo-pregnant ICR mice. This process resulted in the successful generation of live offspring.

### 2.5. Genotyping

Genomic DNA was extracted from mouse tail biopsies via the Quick Genotyping Assay Kit for Mouse Tail (Beyotime, Shanghai, China) and subjected to PCR to verify the insertion of the hSCARB2 gene. PCR screening was performed via specific primer sets 1 and 2 for the hSCARB2 and WPRE genes (Table S1), with genomic DNA from tail samples as the template. The corresponding plasmid was used as a positive control, whereas a negative control (ddH_2_O without a DNA template) was included to ensure the specificity of the amplification.

### 2.6. Western Blotting

Mouse tissues were homogenized in RIPA buffer (APExBIO, Houston, TX, USA) supplemented with 1× protease inhibitor (PI) (Roche, Basel, Switzerland). The lysates were incubated on ice for 15 min and subsequently sonicated until the solution became clear. Total protein concentrations were measured using a BCA protein assay kit (CWBio, Taizhou, Jiangsu, China). Equal amounts of protein were resolved by 10% SDS–PAGE and transferred onto nitrocellulose (NC) membranes. The membranes were blocked for 30 min at room temperature with MinuteBlock blocking buffer (Affinibody, Wuhan, China). For immunodetection, the membranes were incubated with the following primary antibodies: anti-hSCARB2 (1:1000, HPA018014, Sigma, St. Louis, MO, USA) to detect hSCARB2 and anti-β-actin (1:20,000, Cat No. 20536-1-AP, Proteintech, Wuhan, China) as a loading control. After washing, the membranes were incubated with an HRP-conjugated goat anti-rabbit IgG secondary antibody (1:20,000, Cat No. K1223, APExBIO, Houston, TX, USA). The protein bands were visualized via a ChemiDoc Imaging System (Bio-Rad, Hercules, CA, USA) and enhanced chemiluminescence (ECL) reagents (Affinibody, Wuhan, China).

### 2.7. CVA16 Infection

WT and KI mice (10, 21, and 30 days old) were infected with the viruses (1 × 10^7^ PFU) via intracerebral (i.c.) inoculation. After inoculation, the animals were monitored daily for survival and clinical manifestations over a period of 21 days. The onset and duration of all visible changes, such as reduced mobility, limb weakness, paralysis, and death, were recorded. Six hSCARB2-KI mice and six WT mice were sacrificed on days 5, 7, and 10 after infection and the organs or tissues were harvested for viral distribution analysis, histopathology, and immunohistochemistry.

### 2.8. Real-Time PCR

Total RNA was extracted from tissues via TRIzol reagent (Vazyme, Nanjing, Jiangsu, China) according to the manufacturer’s instructions and subsequently converted into cDNA via the Reverse Transcription (RT) PCR Kit (Takara, Shiga, Japan). The synthesized cDNA was analyzed on a CFX96 Touch™ Real-Time PCR Detection System (Bio-Rad Laboratories, Hercules, CA, USA). Real-time quantitative PCR was performed using TB Green Premix Ex Taq™ II (Takara Bio, Inc., Kusatsu City, Japan). Specific primer sets 3 and 4 (Table S2) were used to detect hSCARB2 and mouse GAPDH mRNA.

Quantitative analysis was performed via the One Step PrimeScript™ RT–PCR Kit (Takara Bio, Inc., Japan). The specific sequences for CVA16 primer set 5 and the probe are shown in Table S3. A standard curve (Table S4) correlating cycle thresholds (Cts) with virus copy numbers was established for viral load quantification. The Cq values were converted to TCID by means of a standard curve, and subsequently, TCID was converted to PFU values according to the equation (TCID_50_ × 0.7 = PFU).

### 2.9. Histopathological and Immunohistochemical Staining

Mouse tissue samples were fixed in 10% formaldehyde, dehydrated, embedded in paraffin, and sectioned into 4 μm thick slices for hematoxylin and eosin (H&E) staining. The slides were deparaffinized, rehydrated, subjected to antigen retrieval, and then blocked in 4% bovine serum albumin (BSA) for immunohistochemical analysis. The hSCARB2 protein was detected via an anti-hSCARB2 antibody (HPA018014, Sigma) diluted 1:1000 in PBS containing 1% BSA. CA16 antigens were detected via an anti-enterovirus 71 antibody (MAB979, Merck, Billerica, MA, USA) that cross-reacted with CA16 and was diluted 1:1000 in PBS containing 1% BSA. The slides were washed with PBST and incubated with a goat poly-HRP-conjugated anti-rabbit IgG secondary antibody (Cat# AS040, ABclonal, Woburn, MA, USA) for 35 min at 37 °C. Peroxidase activity was visualized via an Enhanced HRP-DAB Chromogenic Substrate Kit (Tian Gen Biotech, Co., Ltd., Beijing, China). Finally, the stained slides were examined under a light microscope.

### 2.10. Immunofluorescence Staining

Paraffin-embedded tissue sections were deparaffinized and rehydrated using xylene and graded ethanol. Antigen retrieval was performed by heating the slides in citrate buffer (pH 6.0) for 20 min at 95 °C. After antigen retrieval, the sections were incubated with blocking buffer (5% BSA in PBS) for 30 min at room temperature to block nonspecific binding sites. The sections were then incubated overnight at 4 °C with primary antibodies: an anti-GFAP antibody (ab7260, Abcam, Cambridge, UK) for labeling astrocytes and a recombinant anti-Iba1 antibody (ab178846, Abcam, Cambridge, UK) for labeling microglia. Following PBS washes, the sections were incubated with Alexa Fluor-conjugated goat anti-rabbit IgG (Thermo Fisher, Waltham, MA, USA) for 1 h at room temperature. Nuclei were counterstained with DAPI (Sigma–Aldrich, St. Louis, MO, USA). The stained sections were mounted with an antifade mounting medium (ProLong™ Gold, Invitrogen, Waltham, MA, USA, Waltham, MA, USA) and observed under a fluorescence microscope. Representative images were captured for analysis.

### 2.11. Statistical Analysis

All values are expressed as the means ± SD. Statistical analyses were conducted via GraphPad Prism 10 software for Windows. Kaplan–Meier survival curves were compared via the log-rank test. An unpaired Student’s *t*-test was used to compare viral load levels between groups. Means ± SEMs (standard errors of the mean) were graphed and *p* < 0.05 was considered to indicate statistical significance.

## 3. Results

### 3.1. Generation of ROSA26-hSCARB2 KI Mice

**Figure 1:**
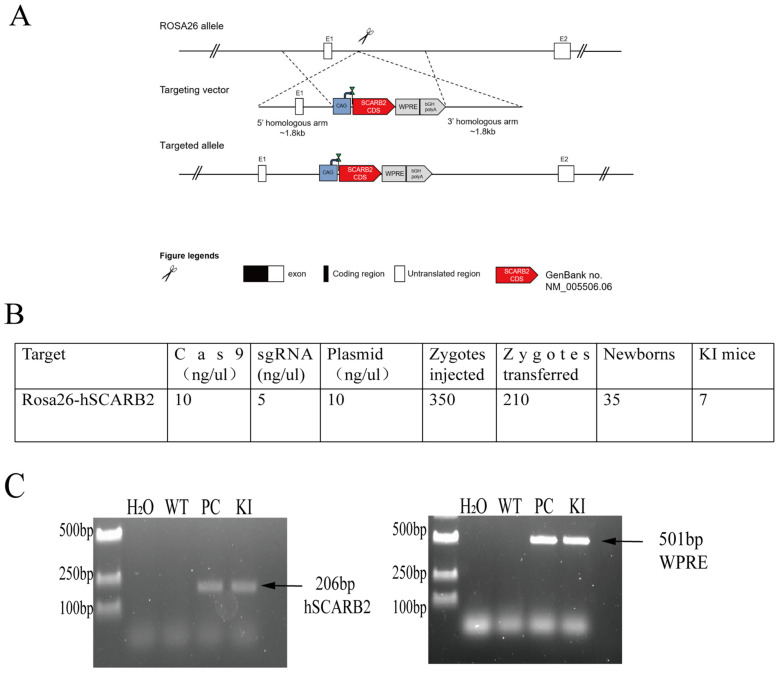
Generation of ROSA26-hSCARB2 knock-in (KI) mice. (**A**) Schematic strategy for creating ROSA26 − hSCARB2 KI mice using CRISPR/Cas9. The CRISPR/Cas9 system was employed to insert an expression cassette containing the CAG promoter, hSCARB2 gene, WPRE element, and PolyA sequence between the homologous arms at the ROSA26 locus. (**B**) The number of embryos injected and transferred, as well as the number of newborns produced during the creation of KI mice using the CRISPR/Cas9 system. (**C**) PCR Genotyping of hSCARB2 knock − in mice. To confirm the presence of the hSCARB2 knock-in, two primer pairs were designed. Primer pair 1 targets the human SCARB2 gene, while primer pair 2 is specific to the WPRE element, ensuring amplification occurs only in hSCARB2 knock-in mice. Plasmid DNA was used as a positive control for PCR. The expected PCR products are 206 bp and 501 bp, respectively.

The CRISPR/Cas9 system was used for homologous recombination to insert a plasmid containing hSCARB2 cDNA into the ROSA26 locus of C57BL/6J mice, yielding hSCARB2-expressing KI mice. The polyA tail and the WPRE element were added to the plasmid to increase the stability of the target fragment’s expression in the mice (Figure 1A). Cas9 mRNA, sgRNA, and the plasmid were coinfected into fertilized C57 mouse eggs. The surviving fertilized eggs were then implanted into pseudo-pregnant ICR mice, resulting in the birth of 35 pups (Figure 1B).

Genomic DNA was extracted from the tails of the pups and analyzed via PCR to verify the genotypes of the mice. The results revealed that 7 of the 35 newborn pups were positive for the hSCARB2 gene, indicating successful generation of F_0_ generation KI mice (Figure 1C). One of the lines was then crossed with C57 mice to obtain the F_1_ generation, named *B6-Gt(Rosa)26Sor^em1(CAG-hSCARB)^* KI mice.

### 3.2. The Expression of hSCARB2 in KI Mice

**Figure 2:**
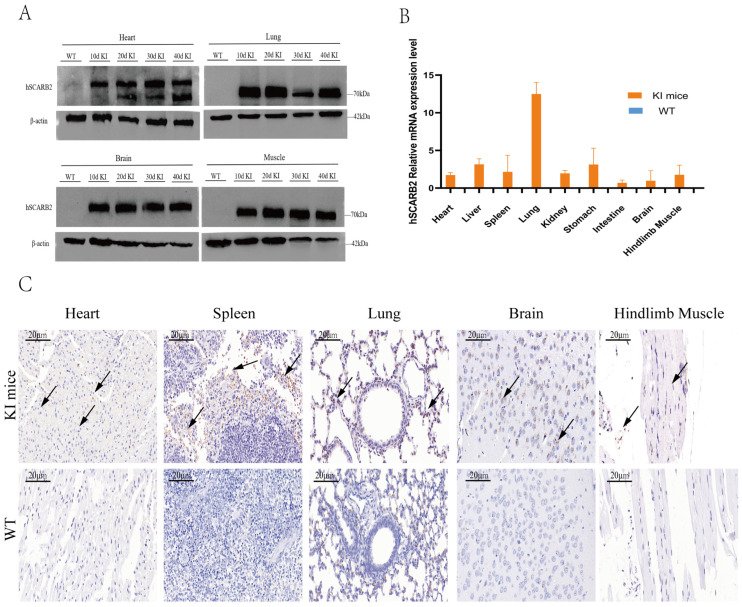
The expression of hSCARB2 protein in F_4_ generation KI mice. (**A**) Western blot analysis was performed to detect hSCARB2 expression in the heart, lung, brain, and hindlimb muscle tissues of mice at different ages, aiming to assess the stability of hSCARB2 expression. (**B**) RT-qPCR analysis of hSCARB2 mRNA expression in KI mice and WT mice at 30 days of age. Data are presented as mean ± SEM from three independent experiments, with normalization to the corresponding GAPDH levels. (**C**) IHC Analysis of hSCARB2 Expression. IHC was performed to analyze hSCARB2 expression in tissues from WT and KI mice at 30 days of age. Sections from the heart, spleen, lung, brain, and hindlimb muscle were stained with an anti-human SCARB2 antibody (brown) and counterstained with hematoxylin for nuclear visualization. The arrows point to hSCARB2 protein expression. Observations were made at 20× magnification, with a scale bar of 20 μm.

To further validate the expression and tissue distribution of hSCARB2 in mice, WB, IHC, and RT-qPCR were performed on F_4_ generation mice. WB results revealed that hSCARB2 protein expression was detected in the heart, lung, brain, and hindlimb muscle tissues of KI mice at 10, 20, 30, and 40 days of age, confirming that the hSCARB2 gene is stably and continuously expressed in these mice (Figure 2A). Similarly, analysis by RT–qPCR revealed a significant increase in hSCARB2 mRNA expression levels in the heart, liver, spleen, lung, kidney, stomach, intestine, brain, and hindlimb muscle tissues of 4-week-old KI mice (Figure 2B). Further analysis by IHC revealed higher levels of hSCARB2 antigen expression in the heart, spleen, lung, brain, and hindlimb muscles of KI mice than in those of WT mice (Figure 2C).

### 3.3. Susceptibility of hSCARB2 Mice to CVA16 Infection

**Figure 3:**
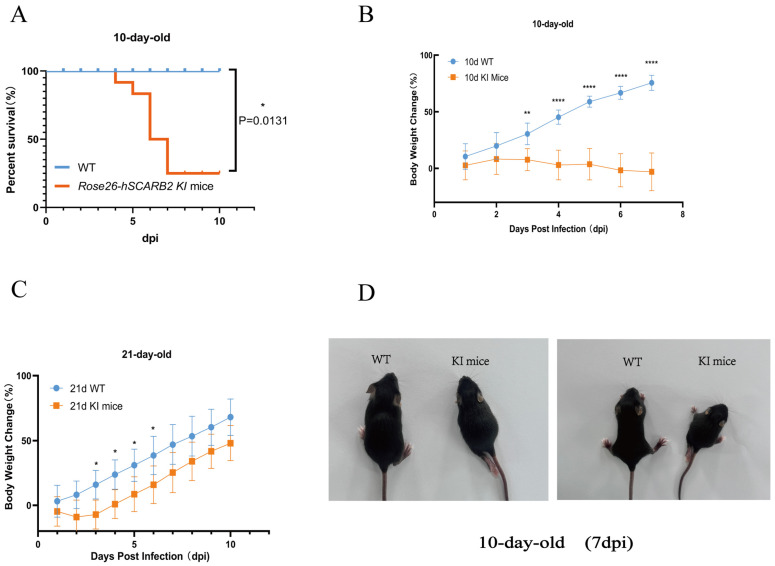
The susceptibility of KI mice to CVA16 infection. (**A**) Survival curves for 10-day-old KI mice and WT mice. Statistical differences in survival rates between CVA16-infected KI mice and WT mice were analyzed using the log-rank test (* *p* = 0.0131). (**B**) Body weight changes were monitored over a 7-day period following CVA16 infection in 10-day-old KI mice (*n* = 7) and WT mice (*n* = 7). Statistical analysis using two-way ANOVA revealed a significant reduction in body weight in hSCARB2 transgenic mice compared to WT mice (**** *p* < 0.0001, ** *p* < 0.01). (**C**) Body weight changes were monitored over a 10-day period following CVA16 infection in 21-day-old KI mice (*n* = 7) and WT mice (*n* = 7). Statistical analysis using two-way ANOVA revealed a significant reduction in body weight in hSCARB2 transgenic mice compared to WT mice (* *p* < 0.05). (**D**) Clinical signs were observed in 10-day-old KI mice following CVA16 infection. These included ataxia, reduced activity, and hind limb paralysis, which typically developed around 6–7 days post-infection (dpi).

To evaluate the susceptibility of hSCARB2 KI mice to CVA16, we intracranially (i.c.) injected 1 × 10^7^ PFU of CVA16 virus into 10-day-old (*n* = 15), 21-day-old (*n* = 15), and 30-day-old (*n* = 15) KI mice, along with wild-type (WT) mice (*n* = 45) and subsequently observed the animals’ conditions and the development of clinical symptoms. Compared with WT mice, 10-day-old hSCARB2 KI mice presented significant weight loss and decreased survival rates (Figure 3A,B). The 21-day-old hSCARB2 KI mice experienced weight loss on day 3 post-infection; however, their weight gradually returned to normal over time (Figure 3C). No significant weight changes were observed in the 30-day-old hSCARB2 KI mice. The clinical symptoms were also most pronounced in the 10-day-old hSCARB2 KI mice. At 6–7 days post-infection (dpi), these mice displayed ataxia, limb paralysis, and mortality (Figure 3D). Of the 15 infected mice, 8 died. In contrast, WT mice of the same age demonstrated a 100% survival rate, with no signs of ataxia or limb paralysis. These results demonstrate that younger hSCARB2 KI mice are more prone to neurological symptoms, which may be associated with age-dependent susceptibility to CVA16.

### 3.4. Dynamic Changes in the CVA16 Viral Load and Detection of Viral Expression in Infected KI Mice

**Figure 4:**
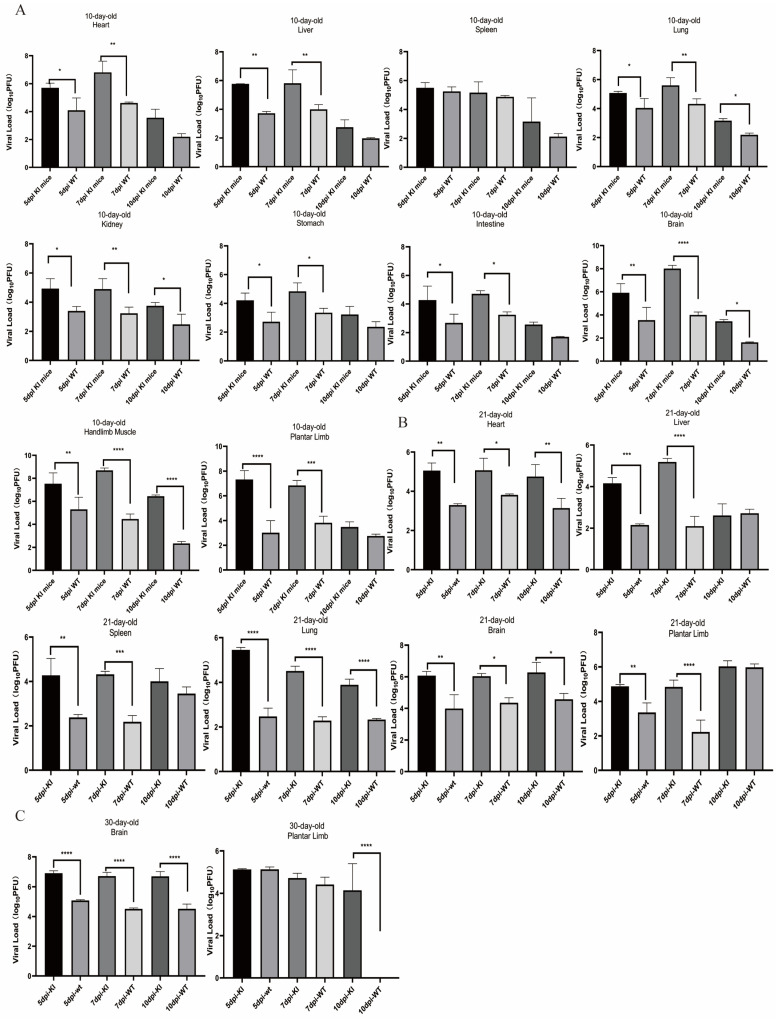

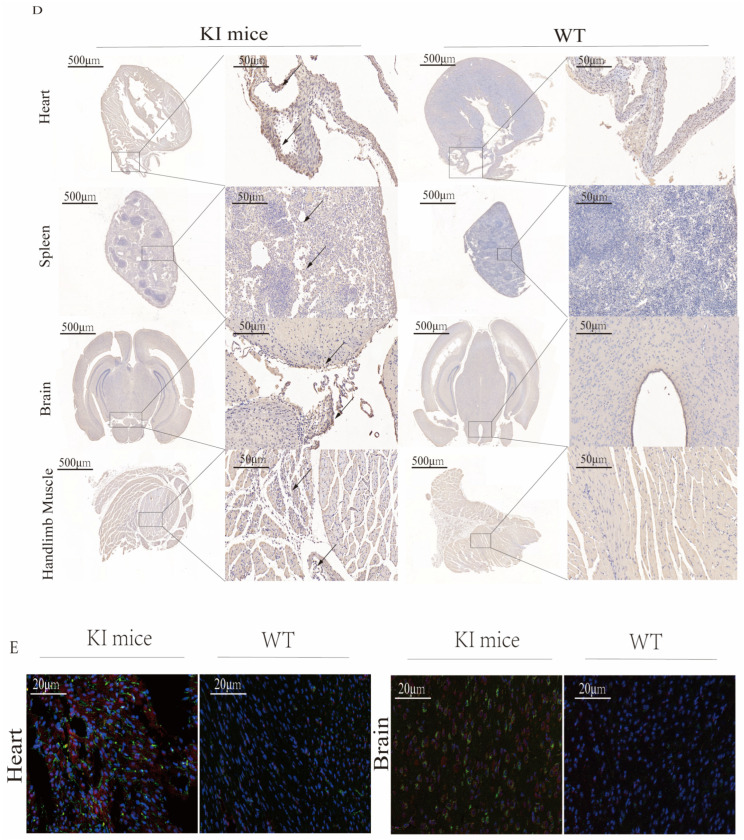
The detection of viral expression in infected KI mice. (**A**–**C**). Quantitative real-time PCR was performed to assess the viral load of CVA16 in ROSA26-hSCARB2 KI mice and WT mice. (**** *p* < 0.0001, *** *p* < 0.001, ** *p* < 0.01, * *p* < 0.05). (**A**) In the 10-day-old mouse group, viral loads in the heart, liver, spleen, lung, kidney, stomach, intestine, brain, hindlimb muscle, and plantar limb of KI mice and WT mice were measured at 5, 7, and 10 days post-infection. (**B**) In the 21-day-old mouse group, viral loads in the heart, liver, spleen lung, brain, and plantar limb of KI mice and WT mice were measured at 5, 7, and 10 days post-infection. (**C**) In the 30-day-old mouse group, viral loads in the brain and plantar limb of mice and WT mice were measured at 5, 7, and 10 days post-infection. Data are presented as the means ± SEM of results from three mice per group. (**D**) Immunohistochemical analysis was performed on 10-day-old mice at 7 dpi to detect CVA16 viral antigens. Representative tissue sections are shown. KI mice exhibited viral antigen-positive areas (indicated by black arrows) in the heart, spleen, brain, and hindlimb muscle. In contrast, no viral antigen was observed in wild-type (WT) control mice. Observations were made at 20× magnification, with a scale bar of 500 μm. (**E**) Viral replication and expression of hSCARB2 in the brain and heart of CVA16-infected mice were examined using immunofluorescence techniques. Ten-day-old KI mice and WT mice were infected via i.c. injection of CVA16 at a dose of 1 × 10^7^ PFU. The animals were euthanized at 7 dpi, and brain and heart tissues were collected. Paraffin-embedded tissue sections of the brain and heart were then stained with specific antibodies and analyzed to assess viral antigens and the expression of hSCARB2. Observations were made at 40× magnification, with a scale bar of 20 μm.

To determine the dynamics of viral replication and tissue distribution after virus inoculation, we performed qPCR to assess the viral load in the heart, liver, spleen, lung, kidney, stomach, intestine, brain, hindlimb muscle, and plantar limb of the mice at 5, 7, and 10 dpi. In 10-day-old hSCARB2 KI mice, viral replication was detected in all tissues examined at 3 dpi, with a significant difference compared with that in the WT group. As the infection progressed, at 7 dpi, the virus had spread from the injection site (brain) to other tissues. The peak viral expression was observed in the heart, liver, and lung of the KI mice. Specifically, the viral load in the heart tissue reached 1 × 10^7^ PFU, that in the brain and plantar limb reached 1 × 10^8^ PFU, and viral replication in the hindlimb muscles was most pronounced, with a viral load of up to 1 × 10^8.5^ PFU (Figure 4A). Similar to 10-day-old hSCARB2 KI mice, high viral loads were observed in the heart, liver, spleen, lung, brain, and plantar limb of 21-day-old hSCARB2 KI mice (Figure 4B). Compared with WT mice, 30-day-old hSCARB2 KI mice presented significant differences in the viral load in the brain and plantar limbs (Figure 4C). Notably, at the mid-infection stage (7 dpi), the highest viral loads were detected in the brain and plantar limbs of all age groups of KI mice, with highly significant differences compared with those in the WT group. These results indicate that, from 10–30 days of age, hSCARB2 KI mice are more susceptible to CVA16 than WT mice are, with the virus showing clear tissue tropism for the brain and hindlimb. Consequently, we speculate that the clinical symptoms of hindlimb paralysis and ataxia observed in the KI mice after CVA16 infection are associated with extensive viral replication in the brain and hindlimb tissues, suggesting that the virus targets the central nervous system (CNS) following infection.

To further validate the tissue distribution of viral antigens in the mice, we selected 10-day-old hSCARB2-KI mice according to the qPCR results. At 7 dpi, we collected heart, spleen, hindlimb muscle, and brain tissues for IHC analysis and performed IF staining on heart and brain tissues. IHC results revealed that the expression of CVA16 antigens peaked in the brain and hindlimb tissues, where the hSCARB2 protein and its mRNA levels were also the highest. In contrast, only a small concentration of viral antigen was detected in WT mice (Figure 4D).

The immunofluorescence results further demonstrated colocalization of the hSCARB2 protein and the CVA16 viral antigen (Figure 4E). This finding further confirms that in hSCARB2 KI mice, the expression levels of viral antigens and viral receptors are aligned after intracranial infection and that both the virus and hSCARB2 exhibit a clear tropism for the brain and hindlimb tissues.

### 3.5. Histopathological Examination of CVA16-Infected Mice

**Figure 5:**
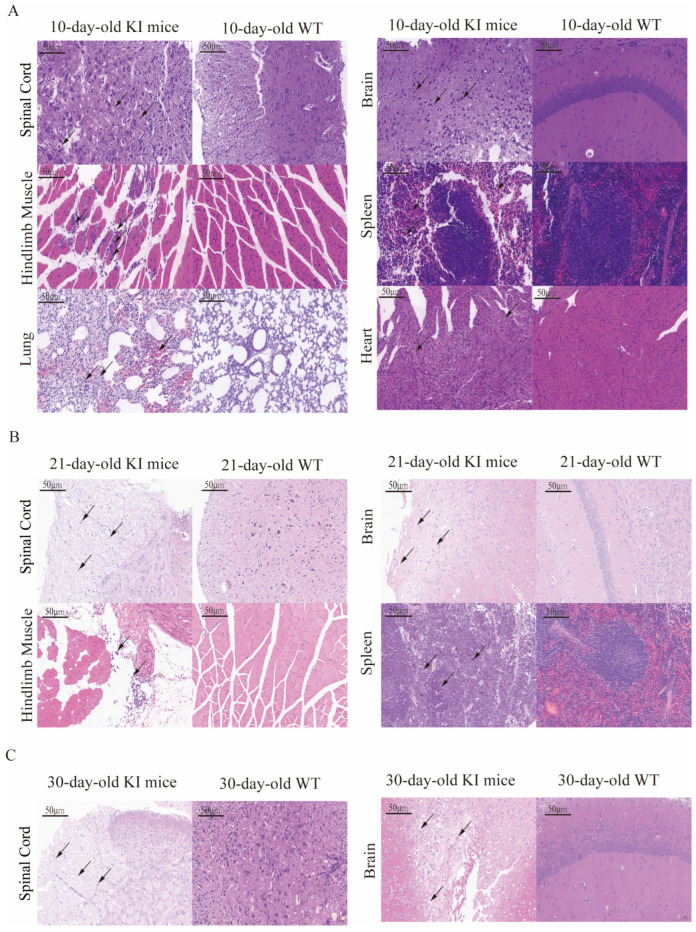
Histopathological analysis was conducted on CVA16-infected mice at 7 dpi. Representative tissue samples from three mice per group, all showing consistent histopathological findings, are presented. Tissue damage (indicated by arrows) was evaluated by a pathologist. Scale bars represent 50 μm. (**A**) The pathological features of CVA16 infection in 10-day-old mice are demonstrated in the spinal cord, brain, hindlimb muscle, spleen, lung, and heart. (**B**) The pathological features of CVA16 infection in 21-day-old mice are demonstrated in the spinal cord, brain, hindlimb muscle, and spleen. (**C**) The pathological features of CVA16 infection in 30-day-old mice are demonstrated in the spinal cord and brain.

To investigate the morphological effects of CVA16 infection on the tissues of hSCARB2 KI mice, tissues of mice infected with CVA16 for 7 days were collected for pathological examination. In 10-day-old hSCARB2-KI mice, at 7 dpi with CVA16, the spinal cord and brain tissues exhibited extensive vacuolar degeneration of neurons, neuronal cell death, disordered cell arrangement, and significant infiltration of microglia. Additionally, neuronal shrinkage, deep staining, unclear nuclear–cytoplasmic boundaries, increased basophilia, and widespread edema were observed in the brain tissue. In contrast, the spinal cord tissue from WT mice showed only a small amount of vacuolar degeneration of neurons and the brain tissue exhibited normal neuronal morphology with no apparent abnormalities. Under the microscope, hindlimb muscle tissue from the 10d hSCARB2 KI-infected group presented localized areas of muscle cell necrosis, disorganized muscle fiber arrangement, and significant lymphocyte infiltration. In contrast, no obvious abnormalities were observed in the muscle tissue of the WT-infected group. Additionally, in 10d hSCARB2 KI-infected mouse group, mild lesions were observed in the spleen, lungs and heart tissues: in the spleen, large areas of hemorrhage were noted in the red pulp, along with abundant neutrophil infiltration; in the lung tissue, extensive necrosis of alveolar and bronchial epithelial cells was observed, accompanied by significant neutrophil infiltration; and in the heart, a small amount of myocardial cell vacuolar degeneration with pale, loose cytoplasm was observed. In contrast, similar tissues in the WT mouse group presented only mild changes or no significant abnormalities (Figure 5A). Pathological examination of the spinal cord, brain, hindlimb muscle, and spleen tissues from 21-day-old hSCARB2 KI mice at 7 dpi revealed significant tissue alterations. In the spinal cord, widespread white matter cell necrosis and edema were observed, with sparse neuronal arrangement and occasional infiltration of small microglia. In the brain, focal areas of cell necrosis were detected, characterized by nuclear fragmentation and dissolution, accompanied by a minor infiltration of microglia. In the hindlimb muscle tissue, a substantial infiltration of neutrophils was noted. The spleen exhibited scattered neutrophil infiltration, with several foci showing pigment deposition. In contrast, no significant changes were observed in the corresponding WT mouse tissues (Figure 5B). Similar pathological changes were observed in the spinal cord and brain tissues of 30-day-old hSCARB2 KI mice at 7 dpi (Figure 5C).

### 3.6. Activation of Glial Cells in CVA16-Infected Brains

**Figure 6:**
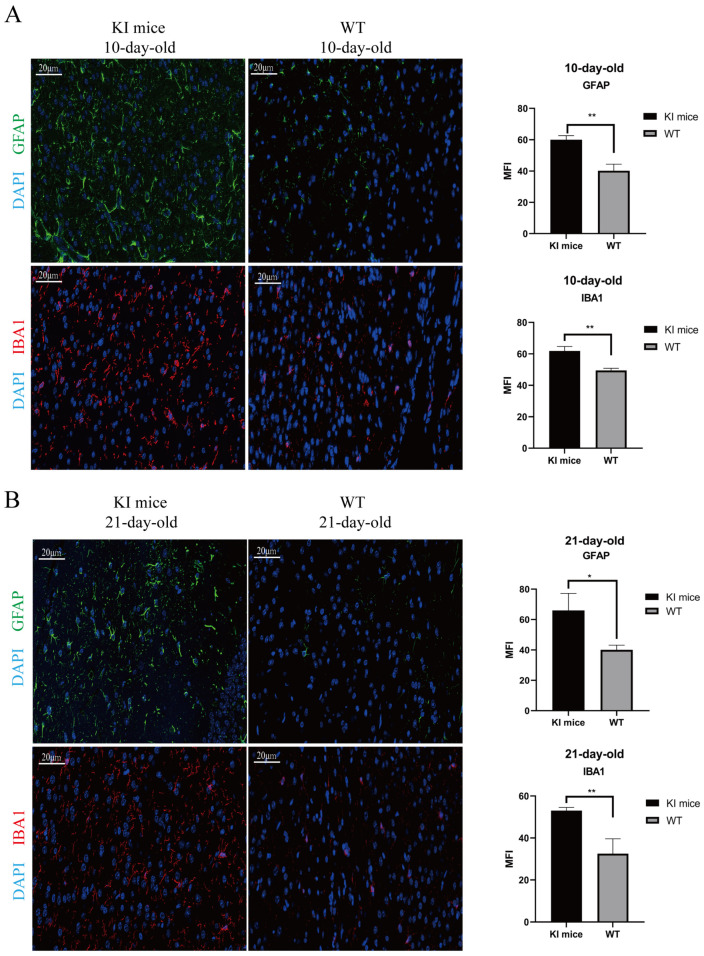
Activation of astrocytes and microglia in brain tissue of KI mice infected with CVA16 at 7 dpi. (**A**,**B**) Paraffin-embedded brain tissue sections from 10-day-old and 21-day-old mouse groups were stained with IBA1 and GFAP to evaluate the activation of microglia and astrocytes. Scale bars represent 20 μm. Data were analyzed using a *t*-test to compare the mean fluorescence intensity (* *p* < 0.05, ** *p* < 0.01).

Given the observed paralysis and mortality in the hindlimbs of the KI mice, we further assessed the extent of CNS damage caused by CVA16 infection. Through IF staining, we found that in the 10- and 21-day-old KI mouse groups, both glial fibrillary acidic protein (GFAP)-positive astrocytes and ionized calcium-binding adapter molecule 1 (IBA-1)-positive microglia were activated in the brain tissues 7 days after CVA16 infection (Figure 6A,B). These findings suggest that these glial cells play crucial roles in virus-induced neuroinflammation. Moreover, these inflammatory cells predominantly accumulate in the cerebral cortex. Therefore, we hypothesize that the extensive activation of astrocytes and microglia in the cerebral cortex is a secondary inflammatory response to the viral invasion of the nervous tissue, which may contribute to the observed ataxia and limb paralysis in mice.

## 4. Discussion

SCARB2 has been identified as a functional receptor for CVA16 entry into host cells and plays a key role in supporting viral infection [28,29]. This study used CRISPR/Cas9 homologous recombination to successfully insert the hSCARB2 gene into the Rosa6 locus of C57BL/J mice, thereby constructing an hSCARB2 knock-in (KI) mouse model. Genetic testing of three consecutive generations confirmed the stability of hSCARB2 gene inheritance and expression, resulting in a stable genetically modified mouse line. Compared with existing hSCARB2 transgenic mouse models [26,30,31], the hSCARB2 KI mouse model developed in this study features an efficient and widespread CAG promoter, along with the addition of WPRE elements and bGH-polyA sequences to increase the transcriptional stability and translation efficiency of hSCARB2. This targeted strategy enabled broad expression of hSCARB2 in mouse tissues and the results were consistent with expectations. In addition, compared with traditional methods of constructing KI mice (such as ESC cloning technology), CRISPR/Cas9 homologous recombination enables the rapid generation of genetically modified mice. Moreover, CRISPR/Cas9 technology is safer than the potential genomic instability associated with ESC cultivation. By modifying the Cas9 protein to prevent DNA double-strand cleavage and the use of specific dual sgRNAs, this technology significantly reduces off-target effects, enhances recombination efficiency, and minimizes or avoids off-target damage in the initial generation of animals [32]. The application of the CRISPR/Cas9 system has ushered in a new era of mammalian genome editing, providing strong support for researchers to construct KI mouse models in a cost-effective and time-efficient manner [33,34].

HFMD is a self-limiting illness that primarily affects children under the age of 5; the severity generally correlates with age [35]. In mice, CVA16 exhibits host restriction, with susceptibility typically limited to neonates under 3 days of age [18]. The selected neonates are usually between 1 and 3 days old, which presents operational challenges. Additionally, the CVA16 neonatal mouse model has limitations, including differences in receptor expression compared with that in humans and the inability to assess vaccine efficacy directly, which hinders the development and application of vaccines [36,37,38]. In this study, we infected 10-day-old hSCARB2 KI mice with 1 × 10^7^ PFU of the CVA16 virus via intracranial injection. Seven days post-infection, the mice presented typical CVA16-like symptoms, such as limb weakness and paralysis, similar to those observed in humans. Notably, this is the first study in which a KI mouse model has been used to successfully simulate human-like clinical manifestations of HFMD following CVA16 infection. Our further investigation revealed that even at 30 days of age, KI mice remain susceptible to CVA16 infection via intracranial injection. qPCR analysis revealed that viral replication peaked at 7 dpi, with the viral load in the brains of 30-day-old mice reaching 1 × 10^7^ PFU, which was significantly greater than that in the brains of wild-type (WT) mice. However, compared with 10-day-old mice, 30-day-old hSCARB2 KI mice did not exhibit typical clinical symptoms, suggesting that host age still influences CVA16 infection. In addition, we found that for 10-, 21- and 30-day-old hSCARB2 KI mice, the viral load and viral antigen expression in various tissues exhibited similar trends: the viral load peaked on day 7 post-infection and gradually declined after day 10. Interestingly, we detected high expression of hSCARB2 in the brain and hindlimb tissues of the mice. After CVA16 infection, high expression of the CVA16 antigen was also observed. This finding provides the first evidence that, in this hSCARB2 KI mouse model, the expression of viral antigens correlates with the expression of the viral receptor following intracranial infection. These findings suggest that hSCARB2 expression may mediate viral entry and replication in mice, increasing their susceptibility to CVA16.

Histopathological analysis revealed varying degrees of damage to the major organs of hSCARB2 KI mice, with particularly severe damage observed in the brain and spinal cord. These tissues exhibited extensive neuronal necrosis and infiltration of inflammatory cells, highlighting the strong neurotropism of the virus. In contrast, previous CVA16 mouse models generated through methods such as intranasal inoculation exhibited weaker viral replication and failed to effectively replicate the typical neurohistopathological features of human HFMD [26]. Therefore, we further explored the pathological mechanisms underlying CVA16-induced CNS damage. Microglia and astrocytes play crucial roles in the inflammatory response within the CNS by locally modulating immune functions [39,40,41]. When homeostasis is disrupted or lost, microglia become activated, resulting in significant morphological and phenotypic changes, including dendritic shortening, cell body enlargement, and increased motility and phagocytic activity [42,43]. In response to environmental changes, astrocytes undergo hypertrophy, upregulate GFAP, exhibit alterations in intracellular calcium levels, activate purinergic receptors, and secrete proinflammatory cytokines [44,45]. These responses collectively contribute to the regulation of neuroinflammation and the formation of pathological mechanisms. In this study, we observed a significant increase in the positive signals of GFAP and IBA1 in the brain tissue of CVA16-infected KI mice through immunofluorescence. This finding indicates a marked activation of glial cells, including astrocytes and microglia, within the brains of KI mice. The activation was predominantly localized to the cortical region, showing a high degree of consistency with the viral load distribution. Pathological examination and fluorescence results revealed that CVA16 infection may induce excessive activation of astrocytes and microglia, leading to an intensified local inflammatory response. This effect, in turn, results in neuronal damage in the cerebral cortex and triggers motor dysfunction characterized by ataxia and limb paralysis, potentially leading to death. These findings suggest that the hSCARB2 KI mouse model we developed offers unique advantages for studying the mechanisms of CVA16-induced infection and the neuropathology of HFMD. However, the exact mechanisms by which viral infection leads to the overactivation of glial cells and their role in central nervous system pathology warrant further investigation.

In conclusion, we successfully constructed an hSCARB2 KI mouse model via the CRISPR/Cas9 system and revealed that 30-day-old mice remain highly susceptible to CVA16 infection. The model’s characteristic widespread expression of hSCARB2 provides a significant advantage in evaluating virus-induced damage across various tissues and organs. Compared with traditional models, this model accurately mimics the tissue pathological changes induced by CVA16 infection and neurologic complications similar to those observed in human infections, including glial cell activation, neuroinflammation, and motor dysfunction, at both the 10- and 21-day-old stages. These findings indicate that the model provides an effective and reliable platform for studying the pathogenic mechanisms and neurotoxicity of CVA16. We anticipate that this model will play a pivotal role in the development of antiviral therapies and the evaluation of CVA16 vaccine efficacy in future studies, showing significant application potential.

## Data Availability

The data supporting the conclusions of this article are included within the article and its supplemental material or are available from the authors upon reasonable request.

## Supplementary Materials

The following supporting information can be downloaded at: https://www.mdpi.com/article/10.3390/v17030423/s1, Table S1: Primers Used in PCR; Table S2: Primers Used in RT-qPCR for detecting hSCARB2 mRNA Expression; Table S3: Probe and Primers used in qPCR for detecting viral load in Mice; Table S4: Standard curve for calculation of lg (TCID).
