# Supplementary material for: A New Human SCARB2 Knock-In Mouse Model for Studying Coxsackievirus A16 and Its Neurotoxicity
Source: Viruses. 2025 Mar 14;17(3):423. doi: 10.3390/v17030423 (PMC11945865; doi:10.3390/v17030423)
Supplement: Supplementary file 1 [file viruses-17-00423-s001.zip › viruses-3504543-supplementary/Supplementary Files/Legend for Tables S1 to S4.pdf]

Table S1. Primers Used in PCR. Set 1: Primers designed for detecting the hSCARB2 gene. Set 2: Primers designed for detecting the WPRE element.

Table S2. Primers Used in RT-qPCR for detecting hSCARB2 mRNA Expression. Set 3: Primers designed for detecting the hSCARB2 gene. Set 4: Primers designed for detecting GAPDH (reference gene).

Table S3. Probe and Primers used in qPCR for detecting viral load in Mice. Set 5: Primers designed for detecting CVA16.

Table S4. Standard curve for calculation of lg (TCID).
