# Supplementary material for: A New Human SCARB2 Knock-In Mouse Model for Studying Coxsackievirus A16 and Its Neurotoxicity
Source: Viruses. 2025 Mar 14;17(3):423. doi: 10.3390/v17030423 (PMC11945865; doi:10.3390/v17030423)
Supplement: Supplementary file 1 [file viruses-17-00423-s001.zip › viruses-3504543-supplementary/Supplementary Files/Table S1.pdf]

Set1F（hSCARB2）	AGAAGGCTGTAGACCAGAGTATCG
Set1R（hSCARB2）	TGCTTTGTTTCTGAGTTCCCTGTAG
Set2F（WPRE）	ACGCTATGTGGATACGCTGCT
Set2R（WPRE）	GATCCGACTCGTCTGAGGGCGAA
