# Supplementary material for: A New Human SCARB2 Knock-In Mouse Model for Studying Coxsackievirus A16 and Its Neurotoxicity
Source: Viruses. 2025 Mar 14;17(3):423. doi: 10.3390/v17030423 (PMC11945865; doi:10.3390/v17030423)
Supplement: Supplementary file 1 [file viruses-17-00423-s001.zip › viruses-3504543-supplementary/Supplementary Files/Table S2.pdf]

Set3F（hSCARB2）	TTATCCATGTTTTCAGGCCCGA
Set3R（hSCARB2）	TGCACTTGTCTGTTATCCACCA
Set4F（GAPDH）	ACTCTTCCACCTTCGATGCC
Set4R（GAPDH）	TGGGATAGGGCCTCTCTTGC
