# Supplementary material for: A New Human SCARB2 Knock-In Mouse Model for Studying Coxsackievirus A16 and Its Neurotoxicity
Source: Viruses. 2025 Mar 14;17(3):423. doi: 10.3390/v17030423 (PMC11945865; doi:10.3390/v17030423)
Supplement: Supplementary file 1 [file viruses-17-00423-s001.zip › viruses-3504543-supplementary/Supplementary Files/Table S3.pdf]

CA16-80 probe	6FAM-ccgccagctcaagtgcagtccc-TAMRA
Set5F（CVA16）	caacccatctgtgtttgtgaaaa
Set5R（CVA16）	ggtatgcactagctggtgacatg
